# The polymorphisms of the *PPARD* gene modify post-training body mass and biochemical parameter changes in women

**DOI:** 10.1371/journal.pone.0202557

**Published:** 2018-08-29

**Authors:** Agata Leońska-Duniec, Pawel Cieszczyk, Zbigniew Jastrzębski, Aleksandra Jażdżewska, Ewelina Lulińska-Kuklik, Waldemar Moska, Krzysztof Ficek, Marta Niewczas, Agnieszka Maciejewska-Skrendo

**Affiliations:** 1 Faculty of Physical Culture and Health Promotion, Szczecin University, Szczecin, Poland; 2 Faculty of Physical Education, Gdansk University of Physical Education and Sport, Gdańsk, Poland; 3 Faculty of Tourism and Recreation, Gdansk University of Physical Education and Sport, Gdańsk, Poland; 4 Faculty of Physiotherapy, The Jerzy Kukuczka Academy of Physical Education in Katowice, Katowice, Poland; 5 University of Rzeszów, Faculty of Physical Education, Rzeszów, Poland; University of Oslo, NORWAY

## Abstract

In this study we examined the genotype distribution of the *PPARD* rs2267668, rs2016520, and rs1053049 alleles in a group of women, before and after the completion of a 12-week training program. There were two significant genotype × training interactions resulting in decreases of total cholesterol (Chol) through training in rs2267668 G allele carriers and significant increases of triglyceride (TGL) levels in rs2267668 AA homozygotes. Carriers of rs2016520 *PPARD* C allele exhibited a significant decrease in Chol through training with an accompanying decrease in TGL. There was also overrepresentation of *PPARD* rs1053049 TT homozygotes in the group with higher post-training TGL levels. Moreover (rs2267668/rs2016520/rs1053049) G/C/T haplotype displayed smaller post-training body mass decrease, suggesting that harboring this specific G/C/T haplotype is unfavorable for achieving the desired training-induced body mass changes. On the other hand, the G/C/C haplotype was significantly associated with post-training increase in fat free mass (FFM) and with lower levels of Chol as well as TGL as observed in the blood of the participants in response to applied training. This observation constitutes the second important finding of the study, implying that when specific training-induced biochemical changes are taken into account, some individuals may benefit from carrying the G/C/C haplotype.

## Introduction

Body Mass index (BMI) as well as other body mass and composition parameters are multifactorial traits that have genetic and environmental components. Today we know that post-training changes of body mass parameters are also controlled by a large number of genes with mild to moderate individual effects–the latest gene-based association analysis generated gene networks associated with the BMI-related sub-phenotypes, including 93 genes associated with BMI phenotypic variability [1]. Many attempts looking for independent BMI associations lead to the suggestion that genes encoding the Peroxisome Proliferator-Activated Receptors (PPARs) are in the group of markers important for the effectiveness of weight-loss strategies. PPARs are nuclear transcriptional factors induced by lipid ligands. The main molecular activity of these receptors is control of the gene expression among PPRE (PPAR response elements) sequences in their regulatory regions. The systemic effect of PPAR induction is transcriptional stimulation (or less often repression) of specific genes, which results in changes of levels of the factors engaged in metabolism of energy substrates (like lipids and carbohydrates). As a consequence of the activity of such proteins, the availability of energy substrates decreases and the ligand-dependent induction of PPARs is reduced–in this way the feedback loop regulates the molecular factors and physiological interaction [2].

So far, three PPAR isoforms have been described, one of them is PPARδ [3], which has been proposed as a key regulator of energy metabolism due to its ability to enhance fatty acid catabolism, energy uncoupling, and insulin sensitivity in multiple tissues [4,5]. In humans, PPARδ is encoded by the *PPARD* gene which is located on chromosome 6 [6]. The structure of the human *PPARD* gene was described by Skogsberg et al. [7]. *PPARD* differs from the classical eukaryotic gene model: it has been reported to encompass 9 exons, of which exons 1–3, the 5'-end of exon 4 and the 3'-end of exon 9, are untranslated [7]. In 2007, five new 5'-untranslated exons (designated exons 2a – 2e) in alternatively spliced transcripts of human PPARδ mRNA were identified [8].

Recent studies have indicated the potential roles for allelic variants in the *PPARD* gene in modulating its mRNA and protein levels which, in turn, affect PPARδ target genes [9–12]. The studies of *PPARD* polymorphisms have been mainly focused on three single nucleotide polymorphisms (SNPs): rs2267668 with its *locus* in intron 3, rs2016520 with its *locus* in the 5’ untranslated region of exon 4, and rs1053049 with its *locus* in the 3’ untranslated region of exon 9. One of them, rs2016520, a T/C transition in nucleotide 15 of exon 4 located 87 base pairs before the start codon, is mostly studied due to its pivotal role in controlling lipid metabolism [4,12]. The functional relevance of rs2016520 SNP comes from its influence on *PPARD* promoter, which in the presence of the rs2016520 C allele is more prone to bind the Sp-1 transcription factor–consequently, the transcription level of the *PPARD* gene rises [13]. The impact of the rs1053049 polymorphic site is connected with control of the plasma low-density lipoprotein cholesterol (LDL-C) levels as well as with regulation of glucose disposal in the skeletal muscles [14]. In studies of the rs2267668 SNP during a 9-month period, Tuebingen Lifestyle Intervention Program revealed that the changes of individual anaerobic threshold as well as insulin sensitivity are dependent on rs2267668 alleles. The rs2267668 G allele was recognized as one of the genetic factors responsible for decreasing aerobic physical fitness. Moreover, *in vitro* analyses showed an effect of lowering the skeletal muscle mitochondrial function in G allele carriers [15].

Taken together, the aforementioned findings suggest that *PPARD* polymorphisms are engaged in developing specific training-induced physiological reactions. In the present study, we have decided to correlate the presence of different *PPARD* alleles with post-training changes of body mass measurements as well as with biochemical parameters of energy metabolism. Looking for any associations, we have examined the genotype and alleles frequencies (described in *PPARD* rs2267668, rs2016520, and rs1053049 polymorphic sites) in female participants engaged in a 12-week training program. Body mass/composition and biochemical parameters were measured before and after the completion of a whole training program.

## Materials and methods

### Ethics statement

The procedures followed in the study were conducted ethically according to the principles of the World Medical Association Declaration of Helsinki and ethical standards in sport and exercise science research. The study was approved by the Ethics Committee of the Regional Medical Chamber in Szczecin (Approval number 09/KB/IV/2011). All participants were given a consent form and a written information sheet concerning the study, providing all pertinent information (purpose, procedures, risks, and benefits of participation). The experimental procedures were conducted in accordance with the set of guiding principles for reporting the results of genetic association studies defined by the Strengthening the Reporting of Genetic Association studies (STREGA) Statement.

### Participants

162 Polish Caucasian women aged 21 ± 1 years (range 19–24) were included in the study. None of these individuals had engaged in regular physical activity in the previous 6 months. They had no history of any metabolic or cardiovascular diseases. Participants were nonsmokers and refrained from taking any medications or supplements known to affect metabolism. Participants were included in a dietary program and on the basis of an individual dietary plan, were asked to keep a balanced diet of approximately 2000 kcal/day. The participants were asked to keep a food diary every day. Weekly consultations were held on which the quality and quantity of meals were analyzed and, if necessary, minor adjustments were made.

### Body composition measurements

All participants were measured for selected body mass and body composition variables before and after the completion of a 12-week training period. Body mass and body composition were assessed using the bioimpedance method as described by Leońska-Duniec et al. [16].

### Biochemical and hematological analyses

Fasting blood samples were obtained in the morning from the elbow vein before the start of the aerobic fitness training program and repeated at the 12th week of this training program (after the 36th training unit). The analyses were performed immediately after the blood collection, as it described earlier [16].

### Training phase

The training stage was preceded by a week-long familiarization stage, when the examined women exercised 3 times a week for 30 minutes, at an intensity of about 50% of their maximum heart rate (HRmax). After the week-long familiarization stage, the proper training started. Each training unit consisted of a warm-up routine (10 minutes), the main aerobic routine (43 minutes), and stretching and breathing exercises (7 minutes). The main aerobic routine was a combination of two alternating styles—low and high impact, as described by Leońska-Duniec et al. [16].

#### Genetic analyses

DNA was extracted from the buccal cells using a GenElute Mammalian Genomic DNA Miniprep Kit (Sigma, Germany) according to the manufacturer’s protocol. All samples were genotyped in duplicate using an allelic discrimination assay on a C1000 Touch Thermal Cycler (Bio-Rad, Germany) instrument with TaqMan® probes. To discriminate *PPARD* rs2267668, rs2016520, and rs1053049 alleles, TaqMan® Pre-Designed SNP Genotyping Assays were used (Applied Biosystems, USA) (assay ID: C__15872729_10, C___8851952_30, and C___8851955_60, respectively), including primers and fluorescently labelled (FAM and VIC) MGB^TM^ probes to detect alleles.

#### Statistical analyses

Allele frequencies were determined by gene counting. An 𝜒2 test was used to test the Hardy-Weinberg equilibrium. To test the influence of *PPARD* rs2267668, rs2016520, and rs1053049 polymorphisms on training response, the mixed 2 × 2 ANOVA with one between-subject factor (genotype) and one within-subject factor (time: before training versus after training) was employed. Additionally, normality Kolmogorov-Smirnov test was used to check for data normality, and post hocTukey test was applied when interaction was significant and was used to perform pair-wise comparisons. Haplotype analysis was conducted with R (https://cran-r.project.org, version 3.1.0) using haplo.stats package and haplo.glm regression function. Percentage change over training was used as the dependent variable, while the *PPARD* haplotypes were used as the independent variables. The level of statistical significance was set at p<0.05.

## Results

*PPARD* genotypes described for rs2267668, rs2016520, and rs1053049 conformed to Hardy-Weinberg equilibrium (p>0.05 in all groups, tested separately). The genotyping error was assessed as 1%, while the call rate (the proportion of samples in which the genotyping provided unambiguous reading) exceeded 95%.

To examine the hypothesis that the *PPARD* rs2267668, rs2016520, and rs1053049 polymorphisms modulate training response, we conducted a mixed 2 × 2 ANOVA for eleven dependent variables (Tables 1–3). There was no main effect of *PPARD* rs2267668 genotypes on dependent variables, although two significant genotype × training interactions, for Chol and triglycerides (TGL) and five significant main effects of training: for fat mass (p = 0.014), FFM (p<0.001), Chol (p = 0.008), HDL-C (p< 0.001), and glucose (p = 0.006) were observed. Carriers of the rs2267668 G allele exhibited a 4.6% decrease in Chol in the course of training (173±24 vs 165±26, p = 0.018 [LSD, Least Significant Difference]), compared with elevation in the AA homozygotes (169±25 vs 170±28, p = 0.633 [LSD]). Similarly, there was a decrease, although not significant, in TGL over the period of training (87±29 vs 77±30, p = 0.075). In addition, TGL rose significantly in the AA homozygotes (78±33 vs 85±37, p = 0.009) (Table 1).

**Table 1:** The *PPARD* rs2267668 genotypes and response to training.

Parameter	*PPARD* rs2267668 genotypes						p values			
	AG (n = 30)		AA (n = 126)		GG (n = 6)		Genotype	Training	Genotype x Training	Genotype x Training GG+AG vs. AA
	Before training	After training	Before training	After training	Before training	After training				
Body mass (kg)	60.83±6.43	61.54±8.72	60.79±8.05	59.97±7.94	61.00±5.47	61.80±4.96	0.950	0.265	0.051	0.331
BMI (kg/m^2^)	21.82±2.25	21.58±2.21	21.62±2.48	21.37±2.44	21.61±2.39	21.88±1.396	0.891	0.332	0.054	0.319
BMR (kJ)	6068±270	6029±268	6065±345	6027±331	6052±204	6028±229	0.998	0.057	0.953	0.942
Fat mass (kg)	23.94±4.84	22.28±5.79	23.93±5.65	22.54±5.69	24.43±4.29	24.96±4.12	0.802	**0.014**	0.091	0.821
FFM (kg)	45.96±2.74	46.52±2.86	45.75±3.32	46.08±3.32	45.90±2.03	47.83±2.64	0.701	**<0.001**	**0.008**	0.054
TBW (kg)	33.61±2.05	34.10±2.24	33.51±2.74	33.53±3.65	33.61±1.48	35.08±1.93	0.668	0.096	0.297	0.191
Chol (mg/dL)	170.76±23.52	164.90±27.29	168.85±24.90	169.73±27.82	184.16±27.16	164.50±20.91	0.831	**0.008**	**0.022**	**0.021**
TGL(mg/dL)	88.26±27.34	75.36±24.53	77.67±32.92	85.41±36.60	80.66±37.15	86.83±52.40	0.983	0.946	**0.008**	**0.005**
HDL (mg/dL)	63.61±15.10	60.39±10.81	64.94±12.34	60.89±13.33	72.55±15.12	62.90±12.12	0.553	**<0.001**	0.415	0.906
LDL (mg/dL)	88.85±21.98	118.09±60.95	88.19±20.18	91.73±23.43	95.78±20.05	84.36±15.55	0.242	0.505	0.159	0.156
Glucose (mg/dL)	74.90±8.77	74.86±12.24	78.26±10.29	75.72±9.80	82.83±5.34	72.50±7.06	0.466	**0.006**	0.080	0.690

Mean±standard deviation; p values (ANOVA) for main effects (genotype and training) and genotype x training interaction; bold p values—statistically significant differences (p < 0.05)

**Table 2:** The *PPARD* rs2016520 genotypes and response to training.

Parameter	*PPARD* rs2016520 genotypes						p values			
	CT (n = 32)		TT (n = 123)		CC (n = 7)		Genotype	Training	Genotype x Training	Genotype x Training CC+CT vs. TT
	Before training	After training	Before training	After training	Before training	After training				
Body mass (kg)	61.35±6.08	60.58±5.97	60.73±8.12	59.90±8.01	59.60±6.21	60.25±6.09	0.898	0.165	0.056	0.286
BMI (kg/m^2^)	21.96±2.18	21.72±2.14	21.59±2.50	21.34±2.46	21.38±2.27	21.60±1.93	0.734	0.203	0.063	0.299
BMR (kJ)	6090±255	6053±256	6062±348	6024±334	5993±241	5972±257	0.782	0.050	0.924	0.812
Fat mass (kg)	24.35±4.68	22.70±5.71	23.85±5.70	22.45±5.73	23.71±4.35	24.32±4.12	0.879	**0.011**	**0.049**	0.702
FFM (kg)	46.12±2.59	46.70±2.66	45.74±3.35	46.077±3.33	45.24±2.54	46.78±3.67	0.723	**<0.001**	**0.038**	0.073
TBW (kg)	33.74±1.94	34.22±2.10	33.51±2.76	33.52±3.68	33.12±1.87	34.31±2.69	0.688	0.129	0.366	0.208
Chol (mg/dL)	170.75±23.065	166.15±28.81	168.48±24.84	169.25±27.47	188.00±26.79	169.28±22.91	0.568	**0.010**	**0.030**	**0.038**
TGL (mg/dL)	89.37±28.16	76.68±24.31	76.98±32.73	85.09±36.98	84.28±35.24	89.00±48.18	0.851	0.991	**0.006**	**0.003**
HDL (mg/dL)	63.82±15.16	62.02±10.99	65.02±12.26	60.59±13.32	69.47±10.03	60.62±12.59	0.886	**0.001**	0.237	0.495
LDL (mg/dL)	88.54±20.15	155.62±56.12	87.88±20.29	91.63±23.13	101.24±23.31	91.02±22.63	0.284	0.493	0.197	0.200
Glucose (mg/dL)	74.28±8.98	74.56±11.99	78.56±10.19	75.88±9.83	80.57±7.72	71.71±6.77	0.259	**0.011**	0.086	0.491

Mean±standard deviation; p values (ANOVA) for main effects (genotype and training) and genotype x training interaction; bold p values—statistically significant differences (p < 0.05)

**Table 3:** The *PPARD* rs1053049 genotypes and response to training.

Parameter	*PPARD* rs1053049 genotypes						p values			
	CT (n = 44)		TT (n = 113)		CC (n = 5)		Genotype	Training	Genotype x Training	Genotype x Training CC+CT vs. TT
	Before training	After training	Before training	After training	Before training	After training				
Body mass (kg)	60.71±6.17	59.77±5.77	60.82±8.24	60.08±8.21	61.34±6.89	61.86±6.72	0.930	0.132	0.147	0.820
BMI (kg/m^2^)	21.75±2.28	21.44±2.16	21.62±2.50	21.40±2.48	21.74±2.30	21.90±1.91	0.949	0.138	0.140	0.631
BMR (kJ)	6056±254	6023±229	6067±354	6028±346	6101±287	6055±318	0.963	**0.034**	0.945	0.815
Fat mass (kg)	23.71±5.04	22.00±5.77	23.98±5.65	11.63±5.64	25.26±4.69	26.56±3.83	0.489	0.100	**0.016**	0.881
FFM (kg)	46.01±2.46	26.45±2.52	45.72±3.44	46.10±3.42	45.62±2.88	47.18±4.24	0.815	**<0.001**	0.129	0.416
TBW (kg)	33.46±2.42	34.03±1.97	33.57±2.67	33.52±3.81	33.40±2.12	34.62±3.09	0.869	0.164	0.261	0.121
Chol (mg/dL)	168.04±22.77	166.36±26.96	169.67±25.25	169.55±27.91	187.40±28.27	168.00±24.28	0.619	**0.034**	0.124	0.344
TGL (mg/dL)	80.97±27.11	73.86±25.02	79.09±34.17	87.75±38.54	83.60±32.34	75.60±18.71	0.513	0.682	**0.019**	**0.005**
HDL (mg/dL)	63.45±14.92	61.23±12.56	65.37±12.22	60.77±13.06	69.30±12.96	60.06±10.95	0.888	**0.002**	0.262	0.370
LDL (mg/dL)	87.42±19.47	108.51±133.41	88.57±20.47	91.76±23.41	99.34±29.19	92.82±27.45	0.532	0.601	0.327	0.212
Glucose (mg/dL)	75.06±9.49	74.04±12.99	78.89±9.97	76.12±9.05	77.40±11.97	72.20±2.94	0.139	0.076	0.535	0.463

Mean±standard deviation; p values (ANOVA) for main effects (genotype and training) and genotype x training interaction; bold p values—statistically significant differences (p < 0.05)

Likewise, there was no main effect of *PPARD* rs2016520 genotypes on dependent variables, however, we observed four significant genotype × training interactions (for fat mass, FFM, Chol, and TGL), as well as the five significant main effects of training: for fat mass (p = 0.011), FFM (p<0.001), Chol (p = 0.010), HDL-C (p = 0.001), and glucose (p = 0.011). Carriers of the rs2016520 C allele demonstrated a 4.6% decrease in Chol in the course of the training (174±24 vs 167±28, p = 0.032 [LSD]), compared with an elevation in the TT homozygotes (168±25 vs 169±28, p = 0.681 [LSD]). As in rs2267668, there were opposite effects of training with regard to TGL. Specifically, carriers of the rs2016520 C allele demonstrated a decrease in TGL (88±29 vs 79±30, p = 0.068), while in the TT homozygotes, it increased significantly in response to training (77±33 vs 85±37, p = 0.006) (Table 2).

There was no main effect of *PPARD* rs1053049 genotypes on dependent variables. We found four significant main effects of training: for BMR (p = 0.034), FFM (p<0.001), Chol (p = 0.034), and HDL-C (p = 0.002). In addition, there were two significant genotype × training interactions (for fat mass and TGL) observed. TGL decreased (not significantly) in carriers of the rs1053049 C allele (81±27 vs 74±24, p = 0.124) and increased significantly in the TT homozygotes (79±34 vs 88±39, p = 0.005) (Table 3).

Analysis of association with haplotypes is presented in Table 4. Carriers of the *PPARD* (rs2267668/rs2016520/rs1053049) G/C/T haplotype had smaller decreases in body mass and BMI (by 2.5% per haplotype copy, p = 0.009 and by 2.3% per haplotype copy, p = 0.008, respectively) as compared with individuals homozygous for the reference A/T/T (frequency 81.7%) haplotype. Carriers of the G/C/C haplotype had significantly greater increase in FFM from training (by 1.0% per haplotype copy, p = 0.041) compared with reference haplotype. Conversely, the G/C/C haplotype was significantly associated with decreased Chol (by 4.3% per copy, p = 0.042) and TGL (by 19.7% per copy, p = 0.003) in response to training. The A/C/C haplotype was associated with an increase in HDL-C from training (by 21.5% per copy, p = 0.015) (Table 4).

**Table 4:** Haplotype-based *PPARD* rs2267668/rs2016520/rs1053049 analysis.

Parameter	A/C/C Hap1 (12.35%)			A/T/C Hap2 (4.06%)			G/C/C Hap3 (11.37%)			G/C/T Hap4 (1.59%)		
	coef	t	p	coef	t	p	coef	t	p	coef	t	p
Body mass (kg)	0.006923	0.558062	0.578	-0.003400	-0.477503	0.634	0.002841	0.676132	0.500	0.024623	2.627470	**0.009**
BMI (kg/m^2^)	0.004060	0.353264	0.724	-0.005550	-0.841703	0.401	0.002635	0.677180	0.499	0.023131	2.666457	**0.008**
BMR (kJ)	0.006196	0.691673	0.490	0.004931	0.960646	0.338	-0.001665	-0.549618	0.583	0.011139	1.651473	0.101
Fat mass (kg)	0.051927	0.974905	0.331	-0.029163	-0.939715	0.349	0.012126	0.667430	0.505	0.008008	0.193325	0.847
FFM (kg)	-0.011401	-0.799745	0.425	-0.003444	-0.413846	0.680	0.010004	2.059366	**0.041**	0.016608	1.492638	0.138
TBW (kg)	-0.004812	-0.112555	0.911	0.034568	1.413016	0.160	0.018910	1.307392	0.193	0.020586	0.641085	0.522
Chol (mg/dL)	0.008839	0.142615	0.887	0.031507	0.882617	0.379	-0.043133	-2.051191	**0.042**	-0.060477	-1.282811	0.201
TGL (mg/dL)	-0.12333	-0.64305	0.521	-0.07159	-0.64564	0.519	-0.19740	-3.03008	**0.003**	0.01634	0.11121	0.912
HDL (mg/dL)	0.214908	2.447421	**0.015**	0.035729	0.710117	0.479	-0.008334	-0.280140	0.780	0.003638	0.055012	0.956
LDL (mg/dL)	-0.163781	-0.413547	0.680	-0.007861	-0.034780	0.972	0.183027	1.366429	0.174	-0.205438	-0.694223	0.489
Glucose (mg/dL)	0.0612840	0.8328114	0.406	-0.0002243	-0.0053067	0.996	0.0033776	0.1354351	0.892	-0.0595107	-1.0686303	0.287

t—t statistics; coef—regression coefficient; bold p values (t-test)—statistically significant differences (p < 0.05)

## Discussion

To address the question of whether the common *PPARD* polymorphisms influence selected body composition measurements as well as lipid and lipoprotein phenotypes, we chose to correlate the distribution of genotype, alleles, and haplotypes described in *PPARD* rs2267668, rs2016520, and rs1053049 polymorphisms in female participants engaged in a 12-week training program. The changes in body mass/composition and biochemical parameters measured before and after training have been analyzed in the context of carrying specific *PPARD* alleles or its haplotype combinations. The results provide some further evidence that *PPARD* plays a role in human lipid and carbohydrate metabolism and, in consequence, in weight control. Our major concern was whether the *PPARD* polymorphisms are involved in the modulation of lipid profile, and if so, to what extent.

When tested individually, our statistical analyses revealed that harboring a specific *PPARD* genotype may be associated with different post-training changes of measured biochemical parameters. With reference to *PPARD* rs2267668 genotypes, there were two significant genotype × training interactions (for Chol and TGL) in which a greater decrease of Chol over training was observed, in the rs2267668 G allele carriers and a significant increase of TGL levels in the AA homozygotes. Moreover, carriers of an rs2016520 *PPARD* C allele exhibited a significant decrease in Chol from training with accompanying decreases in TGL. Another finding in the current study is the overrepresentation of *PPARD* rs1053049 TT homozygotes in the group characterized by significantly higher post-training TGL levels.

Little is known about the molecular mechanism underlying *PPARD* polymorphisms influence on human metabolism and most information comes from animal studies. Increased PPARδ expression in adipose tissue in mice has a protective effect against elevated adiposity and serum lipid levels [17]. *PPARD* transcription is activated in cardiac and skeletal muscles, as well as in adipocytes, providing energy from fatty acid catabolism during starvation and ensuring energy supplies for working muscles, while in a well-fed state the PPARδ level decreases [18]. Animal studies suggest that PPARδ is involved in Chol metabolism, as treatment with PPARδ agonists has been shown to increase plasma HDL-C levels in db/db mice and in obese rhesus monkeys [19,20]. Furthermore, it was proven that GW501516 (the potent selective PPARδ agonist) causes a significant decrease in fasting plasma TGL and LDL-C in obese rhesus monkeys [20]. Moreover, it has been observed that one of the molecular effects of endurance training is activation of PPARδ with an accompanying development and maintenance of the “slow” (type I) phenotype of muscle fibers at the expense of “fast” fibers (type IIB) [5,21,22]. The intense endurance activities were also recognized as factors stimulating the *PPARD* expression [23]. On this basis the conclusion that *PPARD* polymorphisms may be of relevance in shaping athletic performance and proper physiological reaction associated with physical activity seems to be reasonable [24]. Polymorphic forms of the *PPARD* gene have also been associated with genetic proclivity toward obesity [9,25,26], however, subsequent attempts have failed to replicate this finding [4,27,28]. Other reports have associated *PPARD* polymorphisms with the effectiveness of cardiovascular fitness, demonstrating the crucial role of *PPARD* gene variants in mitochondrial function and, in consequence, in weight control [15,29].

Our previous study on the same *PPARD* genetic markers conducted in four different subgroups of elite athletes and a group of sedentary controls revealed that *PPARD* rs2016520 and rs1053049 were individually associated with elite athletic performance [30]. This was in accordance with several reports that examined the association between one of these *PPARD* polymorphisms and athletic performance and/or health-related phenotypes. In Russian athlete studies, it has been suggested that endurance athletes may benefit from carrying an rs2016520 C allele [31]. However, in a group of Israeli endurance athletes [24] as well as in Mount Olympus marathon runners [31] these results were not confirmed. On the other hand, when tested together with the *PPARGC1A* Gly482/Gly482 genotype, the *PPARD* rs2016520 CC genotype seemed to be positively correlated with elite endurance athletes status [24]. Taking into account the results from *in vitro* analyses, showing increased *PPARD* expression in the presence of an rs2016520 C allele [13], it may be assumed that (nevertheless inconsistent results of some studies) the rs2016520 SNP is a very promising candidate for further athlete performance association studies. The molecular evidence suggested that this SNP interferes with the binding of Sp-1 and results in the *PPARD* transcriptional activity changes. *In vitro* studies have shown higher transcriptional activity for the C allele in comparison with the T allele [13]. Described modification of PPARδ protein function induced by the polymorphism in the *PPARD* would decrease the ability of fat oxidation upregulation in working skeletal muscle which affects physical performance [32]. Indeed, the HERITAGE Family Study revealed that rs2016520 CC homozygotes showed the smallest increases in cardiorespiratory fitness measured by maximal oxygen consumption and a lower training response in maximal power output after 20 weeks of endurance training [29]. Furthermore, CC homozygotes were characterized by higher LDL-C level and a propensity towards a higher risk of coronary heart disease (CHD) than subjects homogenous for the T allele [10,12,13,33,34] although observations still exist in diverse populations which are inconsistent with these findings [27,34].

Regarding the subject of the next *PPARD* polymorphic site analyzed in the current study, it was confirmed that among other *PPARD* SNPs, rs1053049 is also associated with maintenance of glucose homoeostasis in skeletal muscles [14]. Moreover, in Tuebingen Lifestyle Intervention Program it was observed that the polymorphism could affect diet and physical activity induced changes in human body composition. The presence of the rs1053049 C allele was associated with changes in overall adiposity, hepatic fat storage, and relative muscle mass [35]. Finally, regarding the final *PPARD* gene SNP rs2267668 analyzed, little is known about the molecular mechanism underlying its influence on human metabolism and function. Nevertheless, it was shown that increases in anaerobic threshold and insulin sensitivity after a long-term aerobic training program were expressed to a lesser degree in carriers of the rs2267668 G allele compared with AA homozygotes, suggesting that *PPARD* rs2267668 genotypes may influence the effectiveness of aerobic exercise training in increasing aerobic physical fitness [15]. Moreover, the role of *PPARD* rs2267668 in the modulation of aerobic fitness was supported by the study of Thamer et al. [35] revealing that the presence of the G allele in an individual’s genotype is associated with a lower increase in relative muscle volume and a smaller decrease in adipose tissue mass and hepatic fat storage [35].

Collectively with the result obtained in the current study, all these studies support our initial hypothesis and suggest that these variants play a role not only in athletic performance, but also in post-training body mass changes as well as modulation of glucose levels and lipid profile. Moreover, when the results obtained in the presented study were incorporated in the complex haplotype analysis, the novel finding was that carriers of the rare *PPARD* (rs2267668/rs2016520/rs1053049) G/C/T haplotype displayed smaller post-training effects, in terms of body mass decrease, in comparison with individuals homozygous for the most common A/T/T haplotype, suggesting that harboring this specific G/C/T haplotype is unfavorable for achieving the desired training-induced body mass measurement changes. On the other hand, the G/C/C haplotype (when compared with reference haplotype) was significantly associated with post-training increase in FFM as well as with lower levels of Chol and TGL observed in the blood of the participants in response to applied training. This observation constitutes the second important finding of the current study, implying that when specific training-induced biochemical changes are taken into account, some individuals may benefit from carrying the G/C/C haplotype. To the best of our knowledge, our research group is the first team to explore the association of *PPARD* rs2267668, rs2016520, and rs1053049 polymorphisms in haplotype combination with post-training body mass and biochemical parameter changes in physically active participants. Therefore, the current results cannot be discussed with direct comparisons to relevant studies from other authors. However, when considering the combined effects of the analyzed *PPARD* polymorphisms, our previous study conducted among athletes and inactive controls revealed that the *PPARD* A/C/C haplotype (rs2267668/rs2016520/rs1053049) was significantly underrepresented in athletes compared to controls, suggesting that harboring this specific haplotype is unfavorable for becoming an elite athlete [30].

The first limitation of almost every genetic association study is a proper number of participants in a study group–we are aware that in our case it could also be a problem and we see the need of replicating our results in another, preferably larger, population. The second issue is the question of whether the analyzed *PPARD* polymorphisms are true causative factors or perhaps only in linkage disequilibrium with variants directly engaged in developing a specific physical trait. Even if the analyzed *PPARD* polymorphisms actually influence the post-training changes observed in active participants, it should be underlined that *PPARD* diversity probably accounts for only a small portion of phenotypic variability, due to the polygenic character of the traits connected with body mass and biochemical parameters measured in our experiment.

## Conclusions

The results of our experiment suggest that *PPARD* genotypes, either individually or in haplotype combination, can modulate training-induced body mass changes, glucose levels, and lipid profile. We have demonstrated that harboring a specific *PPARD* genotype may be associated with different post-training changes of measured biochemical parameters. From the haplotype analysis, we can confirm the hypothesis that being a carrier of the G/C/T haplotype is unfavorable for achieving the desired training-induced body mass measurement changes. Furthermore, our results also suggest that some individuals may benefit from carrying the G/C/C haplotype, as regards the post-training FFM changes and lowering of Chol levels. One of the major aims of exercise genomics is to finally be able to define molecular markers, which by themselves or in combination with other biomarkers would make it possible to predict the benefits from an exercise program or a physically active lifestyle. Understanding the genetic background of physiological processes would have an enormous impact not only on individualization of exercise programs to be more efficient and safer, but would also better contribute to recovery, traumatology, medical care, diet, supplementation, and many other areas [36].

## Supporting information

S1 Table Raw data of genotyping, body mass and biochemical measurements (DOI 10.17605/OSF.IO/8B6UF). (DOCX)
