# Supplementary material for: The polymorphisms of the PPARD gene modify post-training body mass and biochemical parameter changes in women
Source: PLoS One. 2018 Aug 29;13(8):e0202557. doi: 10.1371/journal.pone.0202557 (PMC6114845; doi:10.1371/journal.pone.0202557)
Supplement: S1 Table — (DOCX) [file pone.0202557.s001.docx]

To enhance the reproducibility of our results, we deposit our laboratory protocols in protocols.io: [dx.doi.org/10.17504/protocols.io.rsmd6c6](https://dx.doi.org/10.17504/protocols.io.rsmd6c6).
